# Editorial: Functional epigenetic regulation in metabolic diseases

**DOI:** 10.3389/fendo.2023.1190693

**Published:** 2023-03-28

**Authors:** Zhiqiang Huang, Hengmi Cui

**Affiliations:** ^1^ Medical School, Nanjing University, Nanjing, China; ^2^ Department of Biosciences and Nutrition, Karolinska Institutet (KI), Stockholm, Sweden; ^3^ Jiangsu Innovation Institute for Biomedicine, Nanjing, China; ^4^ Institute of Epigenetics and Epigenomics, Yangzhou University, Yangzhou, China

**Keywords:** epigenetics, gene dysfunction, SNPs, DNA methylation, transcription factor, diseases

Human metabolism is delicately coordinated by transcription networks and disturbance of such process is closely linked to a range of diseases, including metabolic disorders, autoimmune diseases, and cancer (1–3). Transcriptional alterations are tightly controlled in the chromatin level through epigenetic mechanisms such as histone modification and DNA methylation (4). Epigenetic remodeling is essential not only for the fine-tuned transcriptional waves during developmental process but also play crucial roles to define disease-related transcription signatures. Investigation into these events, including the chromatin modification process and the underlying regulatory mechanisms involving transcription factors and coregulators, will provide valuable insights for therapeutic interventions (5).

DNA methylation is one of the most extensively studied epigenetic marks, and its functional role is well established (6). For instance, using a methylated DNA immunoprecipitation chip (MeDIP-chip) technique in cardiovascular disease cohort, Hu et al. found that changes in DNA methylation levels were associated with several VEGFR signaling pathway genes (*VEGFB*, *PLGF*, *FATP3*, *F2R*, *FATP4*), potentiating their roles as disease biomarkers. Additionally, the author also found calcium signaling pathway genes (*PLCB1*, *CAMK1D*, *DRD5*) were anomalous methylated in participants.

DNA point mutations can occur frequently due to genetic and environmental factors, some of which are significantly associated with diseases such as metabolic disorders (2). Hou et al. reported that frameshift mutations in *KASH5* can lead to infertility in both sexes, by disrupting the interaction of KASH5 and SUN1. As a result, the reproductive development is halted. The discovery could potentially serve as a genetic foundation for the molecular diagnosis of conditions such as azoospermia (NOA), diminished ovarian reserve (DOR), and recurring miscarriages. Similarly, missense mutations (N162D) in exon 3 of *FGF23* are closely associated with hyperphosphatemia familial neoplastic calcinosis (HFTC) by hindering the glycosylation of its protein, as introduced by Zuo et al. The authors found that the mutated FGF23 protein has defective glycosylation levels and protein stability. By utilizing resources from online databases, Wang et al. found that mutations in the *PRKAR1A* gene are linked to hyperthyroidism, with high *PRKAR1A* prevalence significantly associated with hyperthyroidism. In another bladder cancer cohort, Qu et al. found that variations (rs17110453, rs1934951, rs1934953, and rs2275620) in the CYP2C8 gene are closely related to bladder cancer. These findings suggest that these genetic variations could potentially be used as a novel biomarker for identifying and preventing bladder cancer in population. These examples demonstrate how functional mutations can have profound effects on protein function and disease outcomes.

Transcription factor inactivation can contribute to metabolic disorders and diseases, as demonstrated by recent studies (7). Specifically, the TBX19 gene is critical for brain cognition (8). The mutation (Glu280Asp) in TBX19 is associated with isolated ACTH deficiency (IAD) and cognitive impairment reported by Charnay et al. Additionally, the study found that mutations in the TBX19 gene can occur at multiple sites, with multiple pathological effects. PPARG2 plays a crucial role in fatty acid metabolism and has a strong association with obesity (9, 10). In a biochemical study, Zhang et al. found that niacin can regulate β-cell lipotoxicity through its effects on GPR109A and PPARG2. Incretin drugs have been found to alleviate these effects, although the underlying mechanism is not yet fully understood. One possibility is that niacin modulates the expression of GPR109A and PPARG2 by modifying the affinity of specific transcription factors. According to a clinical study from Yang et al., a decrease in the GRIM-19 gene expression is correlated with autophagy-related proteins such as BECLIN1, LC3BII/I and BNIP3, which have been linked to recurrent spontaneous abortion (RSA). The underlying mechanism behind this association is believed to be attributed to the activation of immune and inflammation-related pathways in THP-1 macrophages. The examples presented demonstrate the importance of transcription factors in the pathogenesis of diseases and underscore the need for further research on their role in disease development. Therefore, further research on transcription factors and their role in disease development is essential for advancing medical knowledge and developing new therapeutic interventions.

While there have been significant efforts in the field of functional epigenetics, uncovering their roles in diseases remains a challenge. More work is required to map tissue- and disease-relevant epigenetic alterations in the patients and to investigate the regulatory mechanisms underlying such epigenetic signatures. This information will be crucial in developing effective treatments for metabolic diseases, tumors, and innate immune diseases.

## Author contributions

All authors listed have made a substantial, direct, and intellectual contribution to the work and approved it for publication.
